# Sleep in a Gymnasium: A Study to Examine the Psychophysiological and Environmental Conditions in Shelter-Analogue Settings

**DOI:** 10.3390/ijerph13121186

**Published:** 2016-11-30

**Authors:** Koh Mizuno, Kazue Okamoto-Mizuno, Motoko Tanabe, Katsuko Niwano

**Affiliations:** 1 Faculty of Education, Tohoku Fukushi University, Sendai 981-8522, Japan; niwano@tfu-mail.tfu.ac.jp; 2 Kansei Fukushi Research Center, Tohoku Fukushi University, Sendai 989-3201, Japan; kazue@tfu-mail.tfu.ac.jp; 3 Faculty of Health Sciences, Tohoku Fukushi University, Sendai 989-3201, Japan; m-tanabe@tfu-mail.tfu.ac.jp

**Keywords:** sleep, gymnasium, shelter, children

## Abstract

We aimed to examine sleep in shelter-analogue settings to determine the sleep and environmental conditions in evacuation shelters. A summer social/educational event was conducted in an elementary school, wherein children and their parents (*n* = 109) spent one night in the school gymnasium; a total of 15 children and 7 adults completed the study. Data were recording using wrist actigraphy and questionnaires, from two days before the event to two days after the event. During the night in the gymnasium, sleep initiation in the children was found to be significantly delayed, whereas adults did not show any significant change in actigraphic sleep parameters. Although 57% of adults complained of stiffness of the floor, only 7% of children had the same complaint. The nocturnal noise recorded at four locations in the gymnasium showed that the percentage of 1-min data epochs with a noise level >40 dB ranged from 53% to 74% during lights-out. The number of subjects that woke up during the night showed a similar pattern with the changes in the noise level. The changes in sleep might represent event-specific responses, such as to a noisy environment, and the different complaints between adults and children could be useful in shelter management.

## 1. Introduction

In cases of devastating disasters, appropriate disaster/emergency management is essential to mitigate the harmful effects of a disaster, by alleviating its impact on human health, community function, and infrastructure. Disaster/emergency management covers the actions and utilization of resources specific to the period before, during, and after a disaster [1,2]. Several reports have described the facts and lessons learned in terms of disaster/emergency management following large-scale disasters such as the tsunami in Banda Aceh (2004), Hurricane Katrina (2005), and the Bam, Iran (2003), and Great East Japan (2011) earthquakes [3,4,5,6].

Among the actions of disaster/emergency management, the provision of shelters is one of the most important issues for victims who have lost or experienced damage to their homes. The important role of disaster shelters was emphasized in a study wherein fatality rates in areas with and without shelter access after a cyclone hit Bangladesh (1991) were 40% and 3.4%, respectively [7]. However, there are various problems and inconveniences associated with residing in shelters, as it may diverge from ordinary living conditions [6,8,9]. In such settings, it has been reported that the symptoms of preexisting medical conditions, including hypertension, respiratory problems, and diabetes, worsen [8], thus reflecting the accumulated adverse effect of residing in shelters [6]. Recently, Veenema et al. [10] reviewed the literature on the quality of health care services and health outcomes in shelter residents. Although the related literature is limited, the authors had reported that nurse staffing levels and staff preparedness, access to medications/medication management, infection control, referrals, communication, and mental health were important features related to the quality of disaster health care services.

Insufficient sleep is a significant problem in shelters, and has often been anecdotally reported or broadcasted during disasters. The condition of evacuees in shelters is influenced by various factors that disturb sleep, including tension and anxiety (psychological factors), noise, different bedding and clothes, and reduced privacy (environmental factors). Considering that poor sleep is associated with various health problems [11,12], it appears reasonable that insufficient sleep could be a primary cause of the aggravation of the symptoms of preexisting medical conditions and of the increase in the risk of psychiatric diseases, such as depression and post-traumatic stress disorder. However, only a few studies have reported on the sleep parameters in disaster shelters. Based on their experience with the Great East Japan Earthquake in 2011, Kawano et al. [13] demonstrated an association between shelter crowding and the incidence of sleep disturbance, whereas Mizuno et al. [14] reported on the accidentally obtained objective sleep data during the days of the disaster, which indicated severely fragmented sleep in a subject who moved to a shelter. Other studies have also described anecdotal reports of sleep problems and snoring in shelters [15], as well as the effectiveness of using makeshift cardboard beds to reduce stress and the risk of venous thrombotic disorders among evacuees residing in a shelter [16]. In another disaster (Hurricane Gustav in 2008), an investigation of evacuees with special medical needs (via interview) indicated that most slept without difficulty in the shelter [17]. The subjects of those studies were middle-aged [13,16], elderly [13,14,16], or had health problems [17], and to our knowledge, no study has examined sleep in younger subjects, including children, who are suggested to be the most vulnerable victims during disasters [18]. Moreover, although environmental factors such as coldness and noise reportedly disturb sleep in shelters [13,14], no study has examined sleep and measured these environmental parameters (ambient temperature, humidity, and noise).

In the present study, we aimed to examine the sleep and environmental conditions in adults and children who spent one night in a school gymnasium, which represented a shelter-analogue setting. These data were collected during a social and educational event scheduled in an elementary school, which was termed as a “school camp”. Although the condition of the “school camp”, which involved only one night in a gymnasium without any disaster-specific fear or anxiety, was different from that in the case of a disaster, we believe the data of objective and subjective sleep evaluation in both adults and children (by measuring noise, light, ambient temperature, and humidity) may be useful in such shelter-analogue environmental settings. The following two hypotheses were proposed for this investigation:

(1) Children are more vulnerable to sleep disturbance as compared to adults during the night of the school camp.
(2) When a large number of people sleep together in a gymnasium, a noisy environment dominates for long hours throughout the nighttime and is associated with sleep quality.

## 2. Experimental Section

### 2.1. School Camp

**Figure 1:**
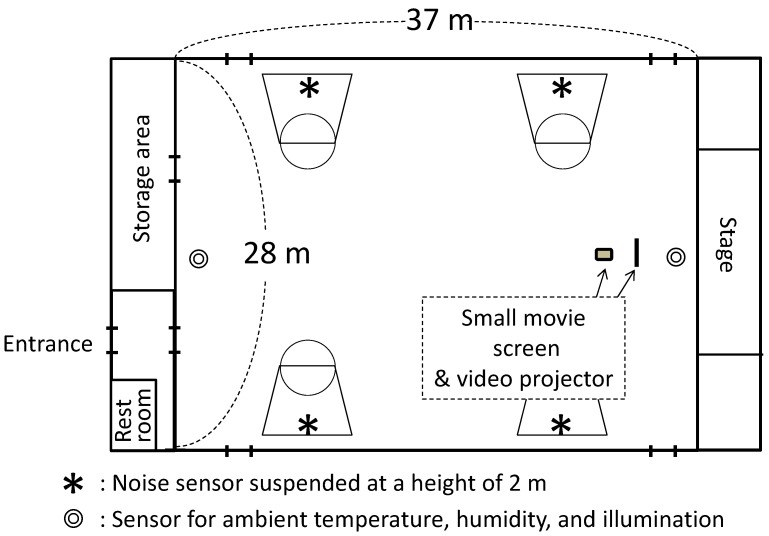
A sketch of the gymnasium where the school camp was conducted.

The school camp was a discretionary social event organized by the parent-teacher association of an elementary school. The school camp was scheduled on weekends in July (early summer), from Saturday afternoon to Sunday morning, and included recreational and educational activities. A total of 109 participants, including pupils of the elementary school and their parents, were assembled at the school at 15:15 on Saturday, stayed overnight at the gymnasium with a floor space of 1050 m^2^ (approximately 28 m × 37 m), and were disbanded at 8:45 on Sunday. Simple meals were provided for dinner and breakfast. Smoking and the drinking of alcoholic beverages were prohibited during the school camp. The scheduled time for sleep was from 21:30 to 6:00. When the lights of the gymnasium were turned off at 21:30, an animated movie was projected for 2 h for children who did not feel sleepy at that time. The small movie screen (2 m × 2 m) was located adjacent to the stage in the gymnasium (Figure 1). The participants slept on the floor of the gymnasium, with sleeping bags that had a polyurethane mattress. For the subjects of the present study, we distributed the same set of sleeping bags and polyurethane mattresses to control for the effect of different bedding.

### 2.2. Subjects

The study subjects were recruited from among the school camp participants. As we sought to only include data from healthy children and adults as the reference sample, we proposed the following exclusion criteria: those with insomnia or snoring, those who were extremely long or short sleepers, those who habitually took daytime naps, those with medical disorders under treatment, or those who had been taking medication. Based on the exclusion criteria, 16 school-aged children, and 7 adults who were parents of the children, and who voluntarily participated, were deemed eligible for the study. Although there were some individuals who snored among the 109 participants, we did not encounter any unhealthy participants or those in bad conditions. The data of one child were lost, due to the unknown termination of the actigraphy measurement, which measures sleep or awake states according to wrist activity. In the final analysis, the data from 15 school-aged children and 7 parents were included. The means and standard deviations (SD) of the physical characteristics for the children and the adults are shown in Table 1. The subjects received a thorough explanation of the study, and provided written consent prior to the start of the measurements. The explanation was provided to the parents as well, and the consent forms were signed by both groups. This study was approved by the ethics committee of Tohoku Fukushi University (project identification code: RS1205285), and was conducted in accordance with the Declaration of Helsinki.

### 2.3. Measurements

Wrist actigraphy was conducted over one week, from three days before to three days after the school camp, by using a Micro Motionlogger (Ambulatory Monitoring Inc., New York, NY, USA). This method measurement is well established for estimating whether the subject is awake or asleep by using a watch-like device that measures wrist activity and can continuously record for 24 h per day over long durations [19]. Hence, the subjects were requested to wear an actigraph on the wrist of their non-dominant hand continuously. As the device is not water-proof and shock resistant, the subjects were asked to remove the device while bathing and performing vigorous physical exercise. For the children, given the risk of loss or physical damage, the actigraph was removed when they went to school on weekdays. In addition, the subjects maintained a written sleep log to track bedtime, waking time, meal times, and times when the actigraph was temporarily removed.

During this period, the ambient temperature, relative humidity, and light levels of the subjects’ bedroom were recorded every 2 min by using a TR 74Ui (T and D Corp., Matsumoto, Japan). The subjects were asked to place the device adjacent to their head while they slept.

During the school camp, similar nocturnal environmental monitoring was conducted in the gymnasium at 2-min intervals for ambient temperature and relative humidity (using RS-12, Espec Mic Corp., Aichi, Japan) and at 5-min intervals for light levels (using LX-1336, Sato Shouji Inc., Kawasaki, Japan). The measurements were conducted from 21:00 to 7:00, and the devices for the measurements were placed at both ends of the gymnasium (Figure 1).

Nocturnal noise in the gymnasium was measured continuously at 1-s intervals by using a noise sensor (CENTER322, Center Technology Corp., New Taipei City, Taiwan). Each noise sensor was suspended from four basketball nets in the gymnasium at a height of 2 m (Figure 1). The measurement period ranged from 10 mins prior to lights-out (21:20) to 10 min after waking (6:10).

A subjective sleep evaluation questionnaire was administered immediately after waking for five days, from two days before to two days after the school camp. For adults, the Oguri-Shirakawa-Azumi sleep inventory MA version (OSA-MA) [20] was administered. The OSA-MA is a standardized questionnaire based on a sample of 670 Japanese adults aged 26–75 years. This questionnaire comprises 16 items estimated on a 4-point scale; accordingly, a T-score is estimated for five factors, including “sleepiness on rising”, “initiation and maintenance of sleep”, “frequent dreaming”, “refreshing”, and “sleep length”. A higher score indicates a better sleep state. The two items of sleep initiation (1, very poor; 2, poor; 3, neither poor nor good; 4, good; 5, very good) and general sleep evaluation (1, very poor; 2, poor; 3, neither poor nor good; 4, good; 5, very good) in the children were recorded by their parents. When the subjects spent one night in the school gymnasium, an additional question was asked, regarding the causes of nocturnal sleep disturbance. The subjects could choose the reasons for sleep disturbance from among the following: noise; stiffness of the floor; coldness of the floor; uncomfortable sleeping bag; uncomfortable pillow; hotness of ambient temperature; coldness of ambient temperature; light; crowdedness of the sleeping quarter; collided against the neighbor or wall; anxiety about sleeping with many persons; and other (free description). These items were determined based on the answers of a preliminary interview with parents and children who had participated in a previous school camp.

### 2.4. Data Analysis

Actigraphic recordings were analyzed using commercial software (Action-W, 2.4.20, Ambulatory Monitoring Inc., New York, NY, USA) to score the sleeping/waking status. The scoring algorithm used for adults and children was different: Cole and Kripke’s algorithm [21] was used for the adults, whereas Sadeh’s algorithm [22] was used for the children. Over the study period, the time in bed (TIB; defined as the primary sleep period during which subjects were trying to sleep in bed) was estimated nocturnally by using the software. If the TIB did not match the results of the sleep log maintained by the subjects, the true TIB was determined by asking the subjects to confirm the actigraph results, sleep log data, and light levels of their bedroom.

Thereafter, bedtime, rising time, sleep latency (SL; time from bedtime to sleep onset), total sleep time (TST; total sleep time during the TIB), wake after sleep onset (WASO; total waking time scored from sleep onset to the end of the last sleep episode in the morning), and sleep efficiency index (SEI; percentage of sleeping time scored during the TIB) were calculated using the software. During the daytime, from rising until bedtime, the total time scored as sleep was calculated as daytime sleep. Since the children did not wear the actigraph when they went to school, daytime sleep in the children was evaluated only on three days (Saturday, Sunday, and the next Monday (a national holiday)).

With regard to the ambient temperature and relative humidity (RH) of the subjects’ bedrooms, the average values during each individual’s TIB was calculated to assess the sleep thermal condition.

Over the seven-day collection period, statistical analysis was performed for data recorded during five days, excluding the first and last days of data collection. Two-way analysis of variance (ANOVA; group: children and adults × day) for repeated measures was used to assess the actigraphic sleep parameters of the subjects’ bedrooms during the TIB. One-way repeated measures ANOVA was used to evaluate the scores of the OSA-MA administered to the adults. The level of significance was considered after the Greenhouse–Geisser correction for repeated measures. Bonferroni tests were used for the pairwise post-hoc comparisons. With regard to subjective sleep evaluation in children, the Friedman test was used to analyze day-to-day variations, whereas Wilcoxon signed-rank tests were used for pairwise post-hoc comparisons. The level of significance was considered to be *p* < 0.05. Statistical analysis was conducted using PASW Statistics 17.0.2 (IBM Corporation, Armonk, NY, USA).

## 3. Results

### 3.1. Actigraphic Sleep Parameters

The results of actigraphic sleep parameters are shown in Table 2. Two-way ANOVA for repeated measures indicated a significant group difference (F (1, 19) = 8.97, *p* < 0.01) and interaction of group × day (F (2.7, 52.1) = 9.19, *p* < 0.001) for bedtime, whereas pairwise post-hoc analysis indicated a significantly delayed bedtime in adults, except during the night of the school camp (*p* < 0.05). During the night of the school camp, although the lights were turned off at 21:30, bedtime for the children was delayed until 23:06 ± 0:14 (mean ± SE), which was significantly later than that for the adults, as well as that for the children before and after the school camp (*p* < 0.05).

In contrast, the rising time showed no group differences, although a significant effect of day was detected (F (4, 76) = 6.38, *p* < 0.01). The scheduled rising time in the morning of the school camp was set at 6:00, and both adults and children showed the earliest rising time during the morning on all days, except for Tuesday (after Monday night) in adults. Post-hoc analyses indicated a significantly earlier rising time for the children on the morning of the school camp (Sunday morning), compared to Saturday and Monday morning (*p* < 0.05). Due to these changes in bedtime and rising time, the TIB and TST showed significant effects of group (TIB: F (1, 19) = 21.10, *p* < 0.001; TST: F (1, 19) = 22.39, *p* < 0.001), day (TIB: F (2.8, 52.4) = 4.88, *p* < 0.01; TST: F (2.3, 44.5) = 8.26, *p* < 0.001), and interaction of group × day (TIB: F (2.8, 52.4) = 9.74, *p* < 0.001; TST: F (2.3, 44.5) = 7.97, *p* < 0.001). During the nights before and after school camp, the TIB and TST were significantly longer for the children than for the adults (*p* < 0.05). During the night of the school camp, a markedly delayed bedtime for the children led to significant decreases in the TIB and TST, compared to that before and after the school camp (*p* < 0.05). The TIB for the children on the night of the school camp was significantly shorter than that for the adults (*p* < 0.05). On the night after the school camp, the TIB and TST for the children showed the greatest values during the data collection period, and post-hoc analysis revealed that the TST at that point was significantly longer than that on the night of the school camp (*p* < 0.05). SL exhibited a significant group difference (F (1, 19) = 11.05, *p* < 0.01), but no significant effect of day and interaction of group × day. The SL was slightly longer for the adults than that for the children, although no significant different was observed on post-hoc analysis. With regard to WASO, only the interaction of group × day was significant (F (4, 76) = 2.75, *p* < 0.05). Although no significant difference was detected on post-hoc analysis, adults showed the longest mean WASO on the night of the school camp. Moreover, SEI indicated significant effects of day (F (4, 76) = 5.39, *p* < 0.001) and interaction of group × day (F (4, 76) = 2.66, *p* < 0.05). In particular, the SEI for adults exhibited the lowest mean value (85%) on the night of the school camp due to the presence of the longest WASO. In contrast, the mean SEIs for the children ranged from 91% to 95%.

Daytime sleep exhibited a significant effect of group (F (1, 19) = 16.88, *p* < 0.001), which indicated a longer daytime sleep time for the adults, compared to that for the children. Although the effect of day was not significant (F (1.5, 30.1) = 2.43, *p* = 0.12), the mean value of daytime sleep was longest after school camp (Sunday) for both adults and children. As some of the subjects did not have any daytime sleep, we only considered daytime sleep that was ≥5 min. The number of such subjects on Saturday, Sunday, and Monday were 5, 5, and 5 among the adults and 8, 4, and 1 among the children, respectively. The mean values of daytime sleep on Saturday and Monday showed a similar tendency in that adults took a longer daytime nap than children (Table 2). In contrast, on the day after the school camp (Sunday), daytime sleep was comparable between the adults (*n* = 5, 90.4 ± 20.5 min) and children (*n* = 4, 96.0 ± 18.1 min).

### 3.2. Subjective Sleep Assessments

The results of the subjective sleep assessments are shown in Table 3. Although different questionnaires were used by adults and children, the results on the morning after the school camp markedly differed between the two groups. The adults showed no significant effect of day-to-day variations in any of the factor scores of the OSA-MA. The mean factor scores on each day were >45, except for the factor score of “initiation and maintenance of sleep” on Saturday (school camp), Sunday, and Monday. In contrast, poor sleep assessment results for the children were noted on the night of the school camp. A significant effect of day-to-day variations was detected in terms of sleep initiation (*χ*^2^ = 21.64, df = 4, *p* < 0.01), and post-hoc analysis indicated that sleep initiation at that point was significantly worse than that recorded at home on the other nights (*p* < 0.05). In addition, the general sleep evaluation results on the night of the school camp were significantly worse than those on Thursday and Sunday (*p* < 0.05).

Table 4 presents the reported causes of sleep disturbance on the night of the school camp. The most common cause among the children (*n* = 15; 66.7%) was noise. The other common causes (reported by >2 children) included uncomfortable sleeping bags (*n* = 4; 26.7%), light (*n* = 4; 26.7%), hotness of ambient temperature (*n* = 3; 20.0%), and an uncomfortable pillow (*n* = 2; 13.3%). The answers of adults differed from those of the children. Although the cause for sleep disturbance was reported to be noise in three of seven adults (42.9%), the most common cause for the adults was stiffness of the floor and an uncomfortable pillow (*n* = 4; 57.1%). The other common causes (reported by >2 adults) included coldness of the ambient temperature.

### 3.3. Ambient Temperature, Humidity, and Light Conditions during the TIB

Due to the early summer season, the conditions of ambient temperature and RH during the TIB were within the comfortable range for sleep. However, the ambient temperature during the TIB on the night of the school camp (22.8 °C) was significantly lower than that recorded in the subjects’ bedroom before and after the school camp (range, 25.0–28.1 °C). The mean nocturnal RH values throughout the data collection period ranged from 64.6% to 75.1%.

The light levels during the TIB before and after the school camp were primarily <1 lux, except for the morning sunlight before the subjects woke up. On the night of the school camp, as the curtains of the gymnasium windows were opened only just before the subjects woke up (6:00), the light levels during the TIB ranged from 0 to 7 lux, except for the value recorded at 6:00.

### 3.4. Nocturnal Noise in the Gymnasium

**Figure 2:**
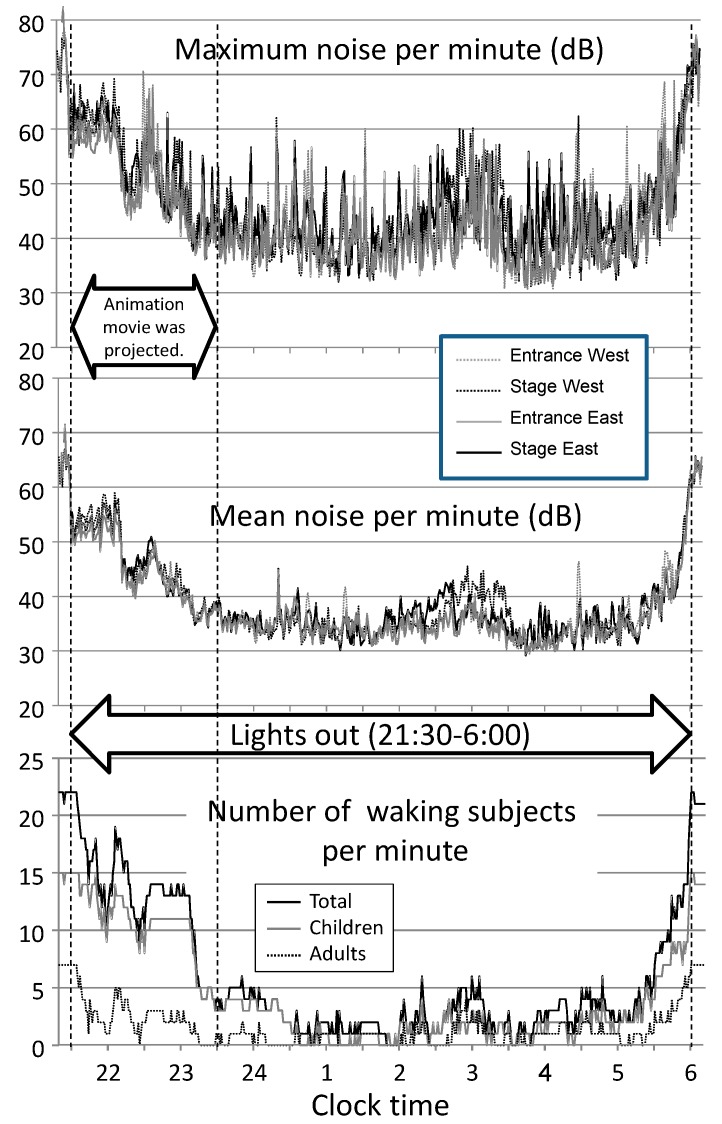
Nocturnal noise in the gymnasium and the number of the subjects who were identified as awake by actigraphy during the time of lights-out.

Figure 2 presents the nocturnal noise measured in the gymnasium and the number of subjects who were identified as waking (by actigraphy) during each minute of lights-out. As the noise was recorded at 1-s intervals, the mean and the maximum noise levels were calculated for each minute to arrange the data resolution similar to that of actigraphy. The changes in noise levels measured at four locations in the gymnasium showed an almost similar tendency throughout the night. The mean noise per minute gradually decreased in the first 2 h after lights-out (21:30 to 23:30, during which an animated movie was projected). Thereafter, a low mean noise level of around 35 dB was maintained until 1 h before the rising time (5:00), except for slight increases from 2:30 to 3:30. After 5:00, the mean noise level gradually increased to >60 dB, which was almost the same level as that before lights-out. During lights-out (21:30 to 6:00), the mean noise levels measured at four locations in the gymnasium during the first 2 h, after the first 2 h, and during the whole time for lights-out ranged from 45 to 47 dB, from 35 to 36 dB, and from 37 to 38 dB, respectively.

As noted in the upper panel of Figure 2 (maximum noise per minute), a transient high noise was frequently recorded throughout the night. The number of 1-min data epochs recording transient noise levels of >40 dB was 377, 307, 297, and 272 (74%, 60%, 58%, and 53% of the total epochs during lights-out) for the four noise sensors. Interestingly, nocturnal noise and the number of waking subjects per minute showed similar patterns throughout the night. Moreover, over the course of the lights-out period, all the 22 participants were almost never simultaneously asleep. The number of minutes during which all the subjects were simultaneously identified as asleep was only 16 min (3.1%) during lights-out. These timings were fragmentarily observed from 0:47 to 3:47, and the longest duration over one continuous time block was 4 min.

## 4. Discussion

To our knowledge, this is the first study to examine sleep and sleep environment under shelter-analogue situations. Nocturnal sleep in evacuation shelters can be disturbed by psychological and environmental factors, and anecdotal reports of poor sleep in evacuation shelters have been described in newspaper articles and broadcasts of various disasters. However, it is difficult to collect comprehensive data on sleep and sleep environment under actual or simulated conditions of evacuation shelters. Although the present study has several limitations and the study shelter conditions differ from those during actual disasters, these data may still be useful as an important reference of the sleep and environmental conditions in evacuation shelters.

Our first hypothesis—children are more vulnerable to sleep disturbance as compared to adults during the night of the school camp—was supported by the results of actigraphic sleep parameters and subjective sleep assessments. In contrast to the lack of any significant change in adults on the night of the school camp, the children showed a significant delay in the actigraphically estimated bedtime, thus resulting in significant decreases in TIB and TST on the night of the school camp. This objectively observed delayed bedtime was accompanied by poor subjective sleep assessment results on the night of the school camp. Husain [18] reported that children are vulnerable to sleep disturbance, nightmares, flashbacks, and re-enactment of traumatic events, following traumatic events such as war, natural disasters, community violence, physical abuse, and catastrophic illnesses. Consistent with this report, children of abused women residing in transitional housing reportedly showed sleep-related behavioral problems [23]. In the present study, although the disaster-specific psychological conditions, such as fear, anxiety, and tension were absent, the psychological excitement related to sleeping together with friends and a feeling of enjoyment might have developed, particularly in the children. As these feelings could also develop in children during similar social and educational events, such conditions would lead to delayed bedtime and poor subjective sleep evaluation results.

Previous studies on poor sleep in evacuation shelters suggest that the reasons for sleep disturbance include reduced privacy and emotional security under hazardous conditions [13], possible percussive noise and the inhalation of dust due to laying down on the floor [16], and coldness in cases where the disaster occurred in early spring [14]. In the present study, the reported causes of sleep disturbance on the night of the school camp showed a different tendency between adults and children. Different tendencies were observed in the items related to discomfort, including bedding, thermal sensation, and environmental light stimulus. Hence, we prepared similar sleeping bags and polyurethane mattresses for both adults and children to control for the effect of bedding. Nevertheless, complaints of stiffness of the floor, uncomfortable pillow, and coldness during sleep were reported at a higher rate from the adults than from the children. In contrast, three (20%) children complained of hotness during sleep, but no adults complained of hotness. The difference in discomfort with the bedding may be attributed to the differences in physical characteristics, such as body weight and joint flexibility, between the adults and children. Moreover, body position shifts during sleep are reportedly less frequent in adults than in children [24]; hence, the longer duration for which pressure is applied on a body part on the floor could be another factor that increases physical discomfort in the adults. Furthermore, a higher metabolic rate and possible difference in the heat dissipation during sleep could be the reasons for the different thermal sensations between children and adults [25]. These findings suggest that the bedding in the shelter should be prepared while considering the differences between adults and children. The reason why only children reported light as a cause of sleep disturbance is poorly understood. The measured light levels suggested that sunlight did not penetrate into the gymnasium during lights-out; hence, one possible reason could be the use of flashlights when participants needed to go to the toilet or when children moved around. This should be controlled for in order to prevent sleep disturbance in actual shelter management.

Our second hypothesis—when a large number of people sleep together in a gymnasium, a noisy environment dominates for long hours throughout the nighttime and is associated with sleep quality—was supported by the results shown in Figure 2. In shelters where many evacuees would sleep together, environmental noise could be a major reason for nocturnal sleep disturbance [26]. Recently, Kawano et al. [13] demonstrated that shelter crowding was associated with sleep disturbance in the case of the Great East Japan Earthquake in 2011, and particularly that noise from neighbors could be a stressor contributing to sleep disturbance. However, to our knowledge, detailed information of environmental noise in shelters or shelter-analogue conditions is lacking. In contrast, the effects of noise on sleep have been widely examined, and have shown that lower noise levels of approximately 33 dB induce physiological reactions such as cortical arousals, body movements, and increased vegetative hormonal secretions [27], and that higher noise levels caused by air traffic induce awakening at levels as low as 48 dB [28,29]. In the present study, the number of 1-min data epochs recording transient noise levels of >40 dB accounted for between 53% and 74% of the lights-out duration for the four respective noise sensors. Furthermore, percussive noise from the floor, which was suggested to be a factor inducing sleep disturbance in a gymnasium, could increase noise stress during sleep. Although the actual noise level in the subjects’ ear was unclear, the results of nocturnal noise level and the answers made by the subjects (43% of adults and 67% of children reported noise as a cause of sleep disturbance) indicated a noisy environment in the gymnasium.

Interestingly, the number of waking subjects in each minute throughout the night showed similar patterns with the changes in the noise level. This result could suggest a potential mutual relationship between environmental noise and the number of waking subjects (i.e., nocturnal noise was made by waking subjects and that nocturnal noise aroused the subjects, and vice versa). As noted in Figure 2, at least one of the 22 subjects was awake during most of the lights-out period. As the total number of the participants residing in the gymnasium was 109, it appeared that there was no time point during the lights-out period at which all the participants soundly slept; this suggests that the waking subjects might make noise that would disturb the sleep of their neighbors. As environmental noise during sleep is known to be associated with various adverse health consequences [27], we believe that instituting measures against nocturnal noise may be a major consideration for evacuation shelter management.

As the present study involved a social and educational event in an elementary school, the differing conditions between the event and those in a disaster shelter should be carefully considered as a limitation of the study. First, as mentioned above, the psychological state of the present subjects was apparently different from that in the case of disasters. Although poor sleep in children may be induced by either positive (happiness or enjoyment) or negative (fear, anxiety, or tension) moods, the psychological state in cases of actual disasters may be more likely to induce poor sleep in the adults. Further, considering the possible situation in disaster shelters where evacuees with medical issues would stay, adverse events due to medical issues might occur during the nights, leading to sleep disturbance in the neighbors. Second, although the length of stay in a disaster shelter generally encompasses several days, the present subjects spent only one night in the gymnasium. Actigraphic recordings obtained during the Great East Japan Earthquake showed severely fragmented sleep in elderly subjects on two nights in the disaster shelter [14]. Subjects who spend several consecutive nights in the gymnasium could experience even more severe effects, possibly due to the accumulation of sleep debt. Moreover, the projection of an animated movie until 23:30 is not performed in disaster shelters. Although the adults’ sleep may not have been influenced by the movie, it could have been a factor that affected sleep in the children. Fourth, although families usually sleep together in a disaster shelter, some children in the present study slept together, and apart from their families. This could represent another factor that induced a delayed bedtime on the night of the school camp. Finally, the results obtained from a small number of the subjects might not be completely generalizable to the conditions in disaster shelters.

Considering the substantial study limitations, we cautiously draw two possible implications for shelter management. First, arrangement and control of environmental conditions are important to prevent sleep disturbance. As it is generally difficult to sleep during the 2–3-h period before habitual bedtime (i.e., “sleep forbidden zone” [30]), the lights-out time (21:30) on the night of the school camp might lead to difficulty in sleep initiation for some adults. Hence, the lights-out time in a shelter should be carefully selected to minimize the gap with individual bedtime and to ensure adequate sleep time. After lights-out, the use of flashlights when evacuees go to the washroom could be a cause for disturbing the sleep of neighbors. Given that aged people generally go to the washroom after sleep onset, the instructions for using flashlights after lights-out and the arrangement of the sleeping location adjacent to the washroom should be given to aged evacuees. In addition, the importance of maintaining silence after lights-out should be publicized in a shelter. Furthermore, age should be considered when distributing beddings. In disaster shelters with limited resources, appropriate distribution should be ensured, while considering that adults tend to feel floor stiffness and children require less bedding than the adults.

## 5. Conclusions

In conclusion, the present study examined sleep and environmental conditions in shelter-analogue settings, by collecting data from children and their parents. The features associated with social and educational events, such as a possible feeling of enjoyment in the subjects and the projection of an animated movie until 23:30, were considered to have induced a significant delay in bedtime and poor subjective sleep evaluation results, which were observed only in the children. Furthermore, the results obtained during a single night of sleep in the gymnasium in a small sample may not be generalizable to actual cases of disaster shelters. On the other hand, the findings related to environmental conditions in the gymnasium and different reported causes of sleep disturbance between adults and children can be applied to shelter management. Future studies examining sleep and environmental stress factors under evacuation conditions are essential, and a practical solution should be developed to reduce stress while considering the effects of the season and the characteristics of the evacuees.

## Figures and Tables

**Table 1:** Physical characteristics of the subjects.

	Age (years)	Height (cm)	Weight (kg)	BMI
Children (6 males and 9 females)	8.5 ± 1.7	133.4 ± 11.3	30.9 ± 10.1	17.0 ± 3.1
Adults (5 males and 2 females)	41.3 ± 4.3	168.4 ± 8.1	71.4 ± 10.4	25.2 ± 3.5

Data are presented as mean ± standard deviation. BMI: body mass index.

**Table 2:** Actigraphic sleep parameters from two days before to two days after the school camp.

	**Children**				
	**Thursday**	**Friday**	**Saturday (School Camp)**	**Sunday**	**Monday (National Holiday)**
Bedtime *^,‡^	21:13 ± 0:19	21:52 ± 0:20	23:06 ± 0:13 ^a,b,c,d^	21:07 ± 0:24	21:35 ± 0:14
Rising time ^†^	6:23 ± 0:07	6:42 ± 0:16	5:39 ± 0:06 ^b,c^	6:49 ± 0:14	6:18 ± 0:18
TIB (min) *^,†,‡^	551 ± 18	530 ± 20	395 ± 15 ^a,b,c,d^	583 ± 23	524 ± 15
SL (min) *	5.4 ± 1.0	7.2 ± 5.7	11.0 ± 3.9	6.9 ± 1.0	5.3 ± 1.5
TST (min) *^,†,‡^	524 ± 13	481 ± 11	362 ± 14 ^a,b,c,d^	548 ± 24 ^e^	478 ± 14
WASO (min) ^‡^	21.7 ± 4.9	42.7 ± 11.5	21.5 ± 8.2	28.3 ± 6.5	40.6 ± 7.8
SEI (%) ^†,‡^	95 ± 1	91 ± 2	92 ± 1	94 ± 1	91 ± 2
Daytime sleep (min) *			8.7 ± 4.7	25.6 ± 13.0	0.9 ± 7.7
Daytime sleep (the subjects slept continuously for ≥5 min, min)			15.6 ± 4.9 (*n* = 8)	96.0 ± 18.1 (*n* = 4)	14 (*n* = 1)
	**Adults**				
	**Thursday**	**Friday**	**Saturday (School Camp)**	**Sunday**	**Monday (National Holiday)**
Bedtime *^,‡^	23:30 ± 0:39 ^f^	23:26 ± 0:45 ^f^	21:57 ± 0:15 ^f^	22:52 ± 0:41 ^f^	22:56 ± 0:26 ^f^
Rising time ^†^	6:09 ± 0:13	6:21 ± 0:24	5:47 ± 0:09	6:34 ± 0:25	5:32 ± 0:42
TIB (min) *^,†,‡^	399 ± 37 ^f^	416 ± 37 ^f^	471 ± 19 ^f^	463 ± 26 ^f^	397 ± 33 f
SL (min) *	8.6 ± 2.5	23.0 ± 13.9	20.0 ± 7.5	6.6 ± 1.1	10.0 ± 3.5
TST (min) *^,†,‡^	367 ± 39 ^f^	352 ± 30 ^f^	403 ± 32	431 ± 28 ^f^	368 ± 29 ^f^
WASO (min) ^‡^	23.9 ± 6.1	45.4 ± 15.8	52.0 ± 17.3	25.4 ± 8.8	21.3 ± 6.7
SEI (%) ^†,‡^	91 ± 2	85 ± 7	85 ± 7	93 ± 7	93 ± 7
Daytime sleep (min) *			33.0 ± 10.1 ^f^	64.7 ± 21.8	54.4 ± 20.4 ^f^
Daytime sleep (the subjects slept continuously for ≥5 min, min)			46.2 ± 8.0 (*n* = 5)	90.4 ± 20.5 (*n* = 5)	75.8 ± 21.8 (*n* = 5)

Data are presented as mean ± standard error. For each column, the bedtime represents the bedtime of subjects on that day, whereas the rising time represents the rising time of subjects on the next morning. TIB: time in bed; SL: sleep latency; TST: total sleep time; WASO: wake after sleep onset; SEI: sleep efficiency index; * Significant effect of group (*p* < 0.01); ^†^ Significant effect of day (*p* < 0.05); ^‡^ Significant interaction of group × day (*p* < 0.05); ^a^ significantly different from the value on Thursday; ^b^ significantly different from the value on Friday; ^c^ significantly different from the value on Sunday; ^d^ significantly different from the value on Monday; ^e^ significantly different from the value on Monday; ^f^ significantly different from the value of the children.

**Table 3:** Subjective sleep evaluation data from two days before to two days after the school camp.

	**Children**				
**Subjective Sleep Evaluation**	**Thursday**	**Friday**	**Saturday (School Camp)**	**Sunday**	**Monday (National Holiday)**
Sleep initiation **	4.2 ± 0.3	3.9 ± 0.3	1.7 ± 0.3 ^a,b,c,d^	4.2 ± 0.2	4.0 ± 0.3
General sleep evaluation	4.7 ± 0.1	3.9 ± 0.3	3.5 ± 0.4 ^a,c^	4.5 ± 0.1	4.0 ± 0.3
	**Adults**				
**OSA Sleep Inventory MA Version**	**Thursday**	**Friday**	**Saturday (School Camp)**	**Sunday**	**Monday (National Holiday)**
Sleepiness on rising	50.2 ± 3.3	47.1 ± 4.5	48.0 ± 3.4	48.6 ± 5.1	48.9 ± 3.8
Initiation and maintenance of sleep	46.8 ± 4.0	46.2 ± 3.2	41.4 ± 5.3	43.1 ± 3.5	41.6 ± 2.3
Frequent dreaming	56.8 ± 1.6	51.4 ± 3.5	57.2 ± 1.2	50.5 ± 3.7	53.8 ± 2.4
Refreshing	49.7 ± 5.2	47.9 ± 3.0	45.3 ± 3.1	46.9 ± 4.9	46.5 ± 3.9
Sleep length	51.1 ± 3.5	46.5 ± 5.1	53.0 ± 2.9	53.0 ± 5.6	49.8 ± 4.1

Data for the adults and the children are presented as mean ± standard error. The values represent those recorded after the corresponding day of the week described in the title line. OSA: Oguri-Shirakawa-Azumi. The significant effect of day-to-day variation was detected with the Friedman test: ** *p* < 0.01; ^a^ significantly different from the value on Thursday; ^b^ significantly different from the value on Friday; ^c^ significantly different from the value on Sunday; ^d^ significantly different from the value on Monday.

**Table 4:** Reported causes of sleep disturbance on the night of the school camp.

	**Noise**	**Stiffness of the Floor**	**Coldness of the Floor**	**Uncomfortable Sleeping Bag**	**Uncomfortable Pillow**	**Hotness of the Ambient Temperature**
Children	66.7 (10)	6.7 (1)	0 (0)	26.7 (4)	13.3 (2)	20 (3)
Adults	42.9 (3)	57.1 (4)	0 (0)	14.3 (1)	57.1 (4)	0 (0)
	**Coldness of the Ambient Temperature**	**Light**	**Crowdedness of the Sleeping Quarter**	**Collided Against the Neighbor or Wall**	**Anxiety about Sleeping with Many Persons**	**Others**
Children	6.7 (1)	26.7 (4)	0 (0)	0 (0)	0 (0)	0 (0)
Adults	28.6 (2)	0 (0)	0 (0)	0 (0)	0 (0)	0 (0)

The values denote percentages of the subjects, with the raw number of subjects enclosed in parentheses.
